# Drivers of AR indifferent anti-androgen resistance in prostate cancer cells

**DOI:** 10.1038/s41598-019-50220-1

**Published:** 2019-09-24

**Authors:** Florian Handle, Stefan Prekovic, Christine Helsen, Thomas Van den Broeck, Elien Smeets, Lisa Moris, Roy Eerlings, Sarah El Kharraz, Alfonso Urbanucci, Ian G. Mills, Steven Joniau, Gerhardt Attard, Frank Claessens

**Affiliations:** 1 Molecular Endocrinology Laboratory, Department of Cellular and Molecular Medicine, KU Leuven, Leuven, Belgium; 2 Division of Oncogenomics, Oncode Institute, Netherlands Cancer Institute, Amsterdam, The Netherlands; 3 Department of Urology, University Hospitals Leuven, Leuven, Belgium; 4 Department of Tumor Biology, Institute for Cancer Research, Oslo University Hospital, Oslo, Norway; 5 Centre for Molecular Medicine Norway, Nordic European Molecular Biology Laboratory Partnership, Forskningsparken, University of Oslo, Oslo, Norway; 6 Centre for Cancer Research and Cell Biology, Prostate Cancer UK/Movember Centre of Excellence for Prostate Cancer Research, Queen’s University, Belfast, UK; 7 Nuffield Department of Surgical Sciences, University of Oxford, John Radcliffe Hospital, Oxford, UK; 8 UCL Cancer Institute, University College London, 72 Huntley Street, London, UK

**Keywords:** Prostate cancer, Cancer models

## Abstract

Inhibition of the androgen receptor (AR) by second-generation anti-androgens is a standard treatment for metastatic castration resistant prostate cancer (mCRPC), but it inevitably leads to the development of resistance. Since the introduction of highly efficient AR signalling inhibitors, approximately 20% of mCRPC patients develop disease with AR independent resistance mechanisms. In this study, we generated two anti-androgen and castration resistant prostate cancer cell models that do not rely on AR activity for growth despite robust AR expression (AR indifferent). They are thus resistant against all modern AR signalling inhibitors. Both cell lines display cross-resistance against the chemotherapeutic drug docetaxel due to *MCL1* upregulation but remain sensitive to the PARP inhibitor olaparib and the pan-BCL inhibitor obatoclax. RNA-seq analysis of the anti-androgen resistant cell lines identified hyper-activation of the E2F cell-cycle master regulator as driver of AR indifferent growth, which was caused by deregulation of cyclin D/E, E2F1, RB1, and increased Myc activity. Importantly, mCRPC tissue samples with low AR activity displayed the same alterations and increased E2F activity. In conclusion, we describe two cellular models that faithfully mimic the acquisition of a treatment induced AR independent phenotype that is cross-resistant against chemotherapy and driven by E2F hyper-activation.

## Introduction

Androgen deprivation therapy is part of the first-line treatment for advanced prostate cancer, but it inevitably leads to the development of castration resistant prostate cancer (CRPC)^1,2^. Interestingly, numerous studies have shown that the androgen receptor (AR) remains a crucial driver in CRPC^3^. The development of novel, highly potent second-generation anti-androgens such as enzalutamide^4^, apalutamide^5^, and darolutamide^6^ has been a major step towards more efficient inhibition of the AR. Enzalutamide is currently the most commonly used second-generation anti-androgen and confers a survival benefit in metastatic and non-metastatic CRPC patients^7–9^. However, the efficacy of enzalutamide and other anti-androgens is limited by the rapid development of acquired resistance and numerous *in vitro* and *in vivo* models have been developed^10–16^. These model systems together with corroborating clinical data from patients have led to the identification of a large variety of anti-androgen resistance mechanisms^17–19^. These involve AR re-activation by mutation of the *AR*, expression of constitutively active splice variants, or intra-tumoural androgen synthesis^18^. In addition, the glucocorticoid receptor may take over the function of the AR (AR bypass) and drive the activity of the AR pathway^16,20^. However, a subpopulation of patients display AR independent mechanisms of anti-androgen resistance that often involve neuroendocrine (NE) trans-differentiation and upregulation of MYCN (also known as N-Myc)^21,22^. Interestingly, the use of the AR signalling inhibitors enzalutamide and abiraterone increased the number of non-neuroendocrine tumours that are independent of AR pathway activity to more than 20% of CRPC patients^23^. Typically, AR independent PCa is associated with loss of AR expression but the groups of Beltran and Sawyers have recently described anti-androgen resistance mechanisms through an AR indifferent state, in which the cells do not depend on AR activity despite continued AR expression^24,25^. The molecular adaptations that lead to such AR indifferent PCa cells remain elusive since the available AR indifferent models systems were generated by targeted methods (cell sorting, knockdown) and not in response to anti-androgen treatment^23,25,26^.

In this study, we describe the independent generation of two distinct multi anti-androgen, castration, and chemotherapy resistant cell lines derived from LNCaP that show an androgen responsive, AR indifferent non-neuroendocrine phenotype that is driven by hyper-activation of the cell-cycle master regulator E2F due to multiple alterations in cyclin D/E, E2F1, RB1 and Myc signalling.

## Results

### Generation of two independent LNCaP derived cell lines with acquired resistance against modern anti-androgens

**Figure 1:**
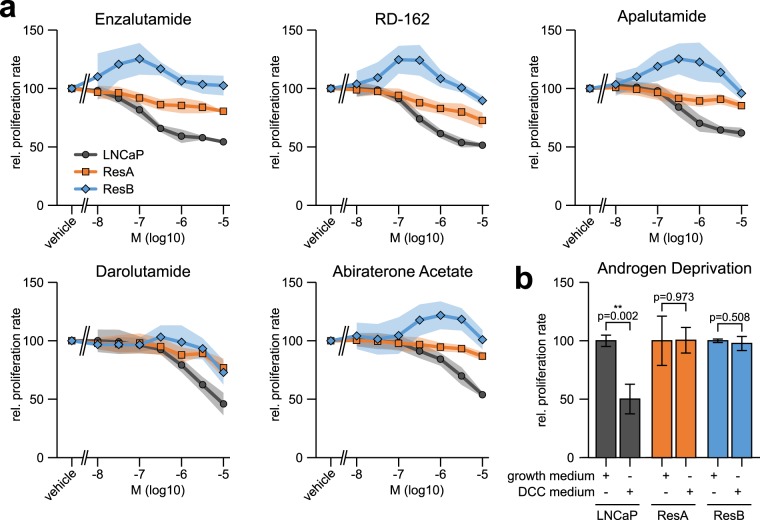
ResA and ResB cells are resistant against modern anti-androgens and androgen deprivation. (**a**) Dose response curves showing the relative proliferation rate at increasing concentrations of the AR inhibitors enzalutamide (MDV3100), RD-162, apalutamide (ARN-509), darolutamide (ODM-201), and abiraterone acetate. All measurements are normalized to vehicle treated cells and set to 100. (**b**) Relative proliferation rate in normal growth medium (containing 10 µM enzalutamide for ResA/ResB) and in androgen deprived medium (dextran-coated charcoal treated serum, DCC). The shaded areas and error bars indicate the 95% confidence interval.

We independently treated the AR reliant prostate cancer cell line LNCaP with enzalutamide (ResA) and RD-162 (ResB) to generate two distinct anti-androgen resistant cell lines. Both long-term anti-androgen treated cell lines were highly resistant against the anti-androgenic effects of enzalutamide, RD-162, apalutamide, darolutamide, and abiraterone acetate (Fig. 1a) as well as against androgen deprivation (Fig. 1b). The IC50 values in LNCaP cells (enzalutamide: 152 nM, RD-162: 354 nM, apalutamide: 465 nM, darolutamide: >1 µM, abiraterone acetate: >1 µM) are in-line with previous reports^4,6^. Of note, the proliferation of ResB cells was stimulated at low concentrations of most anti-androgens. This demonstrates that ResA and ResB cells have acquired a highly resistant phenotype against the most common clinically used therapies targeting the AR pathway.

### Cross-resistance against docetaxel

**Figure 2:**
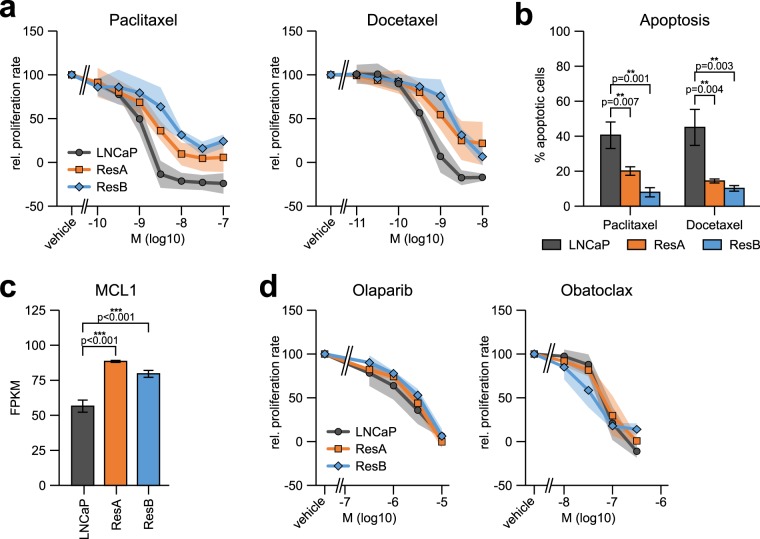
ResA and ResB cells are cross-resistant against docetaxel. (**a**) Dose response curves showing the relative proliferation rate at increasing concentrations of the chemotherapeutic drugs paclitaxel and docetaxel in normal growth medium (containing 10 µM enzalutamide for ResA/ResB). All measurements are normalized to vehicle treated cells and set to 100. (**b**) Caspase 3/7 activity assay showing the percentage of apoptotic cells upon treatment with 10 nM paclitaxel/docetaxel for 48 hours in normal growth medium (containing 10 µM enzalutamide for ResA/ResB). (**c**) FPKM (fragments per kilobase million) mRNA expression of the apoptosis inhibitor MCL1 in conditions similar to their respective growth medium (+10 nM DHT for all cell-lines; +10 µM enz for ResA/ResB). (**d**) Dose response curves showing the proliferation at increasing concentrations of the PARP inhibitor olaparib and the pan-BCL-2 inhibitor obatoclax in normal growth medium (containing 10 µM enzalutamide for ResA/ResB). The shaded areas and error bars indicate the 95% confidence interval.

The group of van Weerden has found that enzalutamide treatment can confer docetaxel cross-resistance^11^, which may impact treatment sequencing in patients. Therefore, we measured the efficacy of paclitaxel and docetaxel on the proliferation of LNCaP, ResA, and ResB cells. Both anti-androgen resistant cell lines were partially cross-resistant against the growth inhibitory effects of both chemotherapeutic drugs, which was due to significantly reduced apoptosis induction in ResA and ResB cells (Fig. 2a,b and Supplementary Fig. 1). Functionally, we found significant upregulation of the anti-apoptotic gene *MCL1* in both anti-androgen resistant cell lines (Fig. 2c). Interestingly, ResA and ResB cells remained sensitive to the PARP inhibitor olaparib and the pan-BCL2 inhibitor obatoclax (Fig. 2d), which may provide promising treatment options for highly therapy resistant patients.

### ResA and ResB cells have a high tumour initiating and self-renewal potential

**Figure 3:**
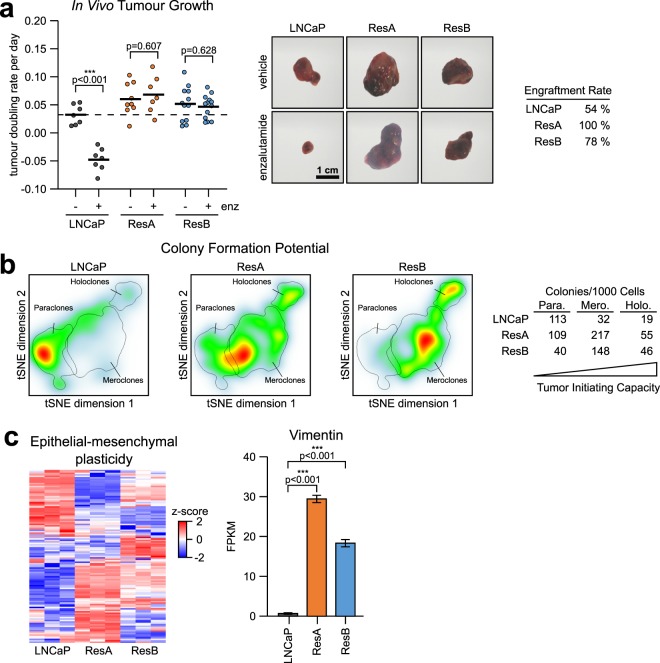
ResA and ResB cells have a high tumour initiating and self-renewal potential. (**a**) Median *in vivo* tumour doubling rates, representative images, and engraftment rates of xenografts derived from LNCaP, ResA and ResB cells in male mice treated with 10 mg/kg enzalutamide or vehicle. The dashed line represents the median of vehicle treated LNCaP tumours. (**b**) Heat maps of high-resolution colony formation assays showing the formation of paraclones (low tumour initiating capacity), meroclones (intermediate) and holoclones (high tumour initiating capacity) in the cell lines in normal growth medium (containing 10 µM enzalutamide for ResA/ResB). (**c**) Heat map of the MSigDB Hallmark “Epithelial Mesenchymal Transition” gene signature expression and FPKM (fragments per kilobase million) mRNA expression of the mesenchymal marker VIM (Vimentin) in the cell lines in conditions similar to their respective growth medium (+10 nM DHT for all cell-lines; +10 µM enz for ResA/ResB). The error bars indicate the 95% confidence interval.

To confirm the anti-androgen resistant phenotype *in vivo*, we performed xenograft experiments in nude athymic mice. Tumours derived from ResA and ResB cells were completely resistant to enzalutamide and their median tumour doubling as well as engraftment rates were higher than in LNCaP cells (Fig. 3a, Supplementary Fig. 2a). Because the tumour initiating potential of individual cells is very variable in cell lines, we quantified the formation of para-, mero-, and holoclones using high-resolution colony formation assays. In contrast to LNCaP, both resistant cell lines readily formed colonies in presence of enzalutamide (Supplementary Fig. 2b). Importantly, the number of colonies associated with high tumour initiating/self-renewal capacity (mero- and holoclones)^27,28^ was elevated in both anti-androgen resistant cells under normal growth conditions (Fig. 3b). In line with the increased clonogenic potential, we observed a high plasticity (>50% significantly deregulated genes) in the Hallmark “epithelial-to-mesenchymal transition” (EMT) gene signature (Fig. 3c) and a 30–40 fold upregulation of the mesenchymal marker *VIM* (also known as Vimentin). In addition, the spatial distribution and morphology (Supplementary Fig. 2c,d) of ResA and ResB cells was substantially altered compared to each other and LNCaP cells. Taken together, this demonstrates that ResA and ResB cells are distinctly different from each other and have an aggressive phenotype with altered morphology/EMT signature.

### ResA and ResB cells have acquired an AR indifferent phenotype

**Figure 4:**
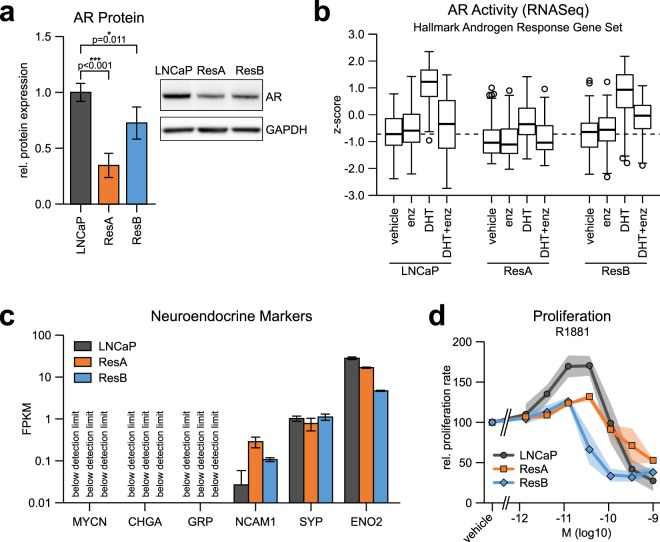
ResA and ResB cells have acquired a non-neuroendocrine AR indifferent phenotype. (**a**) AR protein expression measured by western blotting under normal growth conditions. The uncropped blots are shown in Supplementary Fig. 5. (**b**) Standardized gene expression (z-score) of the MSigDB Hallmark “Androgen Response” signature upon treatment of the cells with 10 nM DHT, 10 µM enzalutamide (enz), combination of the two or vehicle for 18 hours. The dashed line represents the median of vehicle treated LNCaP. (**c**) Log scaled FPKM (fragments per kilobase million) mRNA expression of the neuroendocrine markers MYCN, CHGA, GRP (bombesin), NCAM1 (CD56), SYP, and ENO2 in the cell lines in conditions similar to their respective growth medium (+10 nM DHT for all cell-lines; +10 µM enz for ResA/ResB). (**d**) Dose response curves showing the relative proliferation rate in androgen deprived (DCC) medium supplemented with increasing concentrations of the highly potent synthetic androgen R1881. All measurements are normalized to vehicle treated cells and set to 100. The shaded areas and error bars indicate the 95% confidence interval.

Since LNCaP cells depend on AR activity, we characterized the alterations in AR signalling that enable ResA and ResB cells to grow in presence of enzalutamide. Surprisingly, AR protein expression was significantly reduced in both anti-androgen resistant cell lines (Fig. 4a) and nuclear AR localization was very low in ResA and ResB cells in normal enzalutamide containing growth medium (Supplementary Fig. 3a). We did not detect expression of the constitutively active AR-V7 splice variant, mutations of the AR, or induction of GR expression (Supplementary Fig. 3a–d). In line with this, the transcriptional AR activity was very low in ResA and ResB cells in presence of enzalutamide and during androgen deprivation (Fig. 4b, Supplementary Fig. 3e), suggesting a mechanism of resistance that does not involve global AR reactivation. Neuroendocrine differentiation is frequently associated with development of AR independence but there was no induction of a neuroendocrine signature in ResA and ResB cells (Fig. 4c). Surprisingly, both anti-androgen resistant cell lines were still transcriptionally responsive to androgen treatment (DHT only treatment, Fig. 4b). The magnitude of response in ResB cells was similar to LNCaP, whereas ResA cells had a much weaker response, which is in line with their low AR protein expression (Fig. 4a). As for the well-known biphasic androgen control of proliferation in LNCaP cells^29^, ResA and ResB cells are less responsive to the growth promoting effects of low/intermediate androgen levels (Fig. 4d). R1881 treatment only led to a 30% increase in the proliferation rate of ResA and ResB cells at the maximum, whereas the growth rate of LNCaP cells was almost doubled. Of note, the anti-proliferative effects of high androgen concentrations were preserved since supraphysiological levels of the highly potent androgen R1881 strongly inhibited the growth of all cell lines (Fig. 4d). In the case of ResB, the switch from growth promoting to growth inhibiting responses was shifted to lower R1881 concentrations (10 pM) compared to LNCaP and ResA cells (40 pM). Taken together, both anti-androgen resistant cell lines express the AR and retain a certain level of androgen responsiveness but do not depend on AR activity for growth. This phenotype has been termed “AR indifferent” by other groups^24,25^.

### AR indifferent growth is associated with increased E2F activity

**Figure 5:**
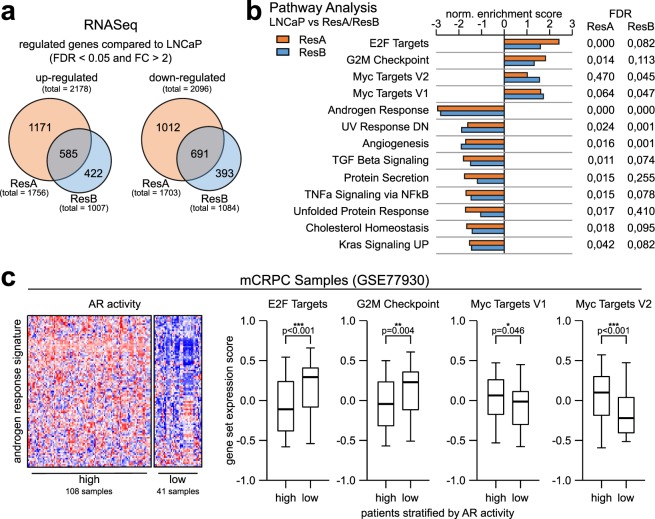
Increased E2F activity drives the proliferation of PCa cells with low AR activity. (**a**) Venn diagram of the significantly up- and down-regulated genes (LNCaP vs. ResA/ResB, FDR < 0.05 and fold change >2) in conditions similar to their respective growth medium (+10 nM DHT for all cell-lines; +10 µM enz for ResA/ResB). (**b**) GSEA pathway analysis (MSigDB HALLMARK gene set) of ResA/ResB cells compared to LNCaP cells in conditions similar to their respective growth medium showing all pathways with a FDR < 0.05 in at least one condition. (**c**) Heatmap and boxplots of the given MSigDB Hallmark signatures in the publicly available GEO dataset GSE77930 stratified by unsupervised clustering into samples with high and low AR activity.

To identify the molecular alterations that enable the AR indifferent growth, we started with RNASeq transcriptome analysis of LNCaP, ResA, and ResB cells. Interestingly, only 30% of all significantly deregulated genes in ResA and ResB cells were found in both resistant cell lines (Fig. 5a and Supplementary Fig. 4a), which underlines the independent nature of ResA and ResB cells. To identify the drivers of the resistance we performed GSEA pathway analysis (Fig. 5b and Suppl. Table 1). Importantly, several cell-cycle related pathways (“E2F targets”, “G2M checkpoint”, “Myc targets”) were significantly enriched in the anti-androgen resistant cell lines. To confirm the clinical relevance of these pathways in PCa with low AR activity we evaluated a publicly available dataset^30^ with 149 mCRPC tissue samples. We classified the samples based on the Hallmark “androgen response” signature by unsupervised clustering into two groups with high (73%) and low (27%) AR activity (Fig. 5c). Similar to the AR indifferent ResA and ResB cells, mCRPC samples with low AR activity had significantly increased expression scores for the “E2F targets” and “G2M checkpoint” signatures, whereas the “Myc targets” signatures showed opposite activity in patient and cell line samples. Importantly, the “E2F targets” and “G2M checkpoint” signatures are partially overlapping and the effects were predominantly caused by elevated E2F activity (Supplementary Fig. 4b). Taken together, this suggests that AR indifferent growth is facilitated by increased activity of E2F and subsequent induction of the pro-proliferative E2F target genes.

### Reprogramming of the cell-cycle master regulators E2F1/RB

**Figure 6:**
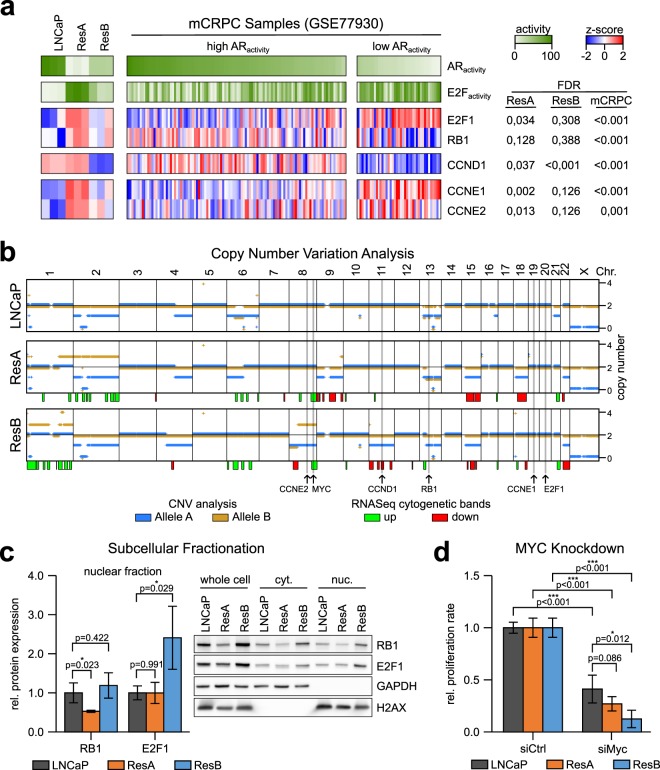
Reprogramming of the cell-cycle master regulators E2F1/RB1. (**a**) AR and E2F gene signature activity and expression of cyclin D/E genes associated with RB1 inactivation in the cell lines and the publicly available GEO dataset GSE77930. (**b**) Copy number variation (CNV) analysis and cytogenetic bands with significantly deregulated mRNA expression in ResA and ResB cells compared to LNCaP. (**c**) Measurement of nuclear protein levels of the cell-cycle activator E2F1 and its negative regulator RB1 in normal growth medium (containing 10 µM enzalutamide for ResA/ResB). The uncropped blots are shown in Supplementary Fig. 5. (**d**) Proliferation of LNCaP, ResA, and ResB upon siRNA mediated MYC knockdown. The error bars indicate the 95% confidence interval.

In normal cells, the transcriptional activity of E2F is predominantly regulated by phosphorylation of the negative regulator RB1 by cyclin D-CDK2/4 (mono-phosphorylation, activates RB1 and suppresses E2F activity) and cyclin E-CDK6 (hyper-phosphorylation, inactivates RB1 and leads to E2F target gene expression)^31^. We observed significant upregulation of CCNE1 and CCNE2 (cyclin E) in ResA cells and downregulation of CCND1 (cyclin D) in ResB cells (Fig. 6a). Importantly, we found the same significant alterations in cyclin D/E also in mCRPC samples with low AR activity. To identify whether the altered mRNA expression of these genes is caused by genomic alterations in the cell lines we performed copy number variation (CNV) analysis coupled with cytoband-specific gene set enrichment analysis (Fig. 6b). We did not observe alterations of CCNE1/2 but one copy of chromosome 11 was lost in ResB and might thus explain the reduced mRNA expression of CCND1 in this cell line. In addition, the mCRPC samples with low AR activity had increased expression of E2F1 and loss of RB1 expression, but we only found minor and mostly non-significant effects in ResA and ResB cells on mRNA level (Fig. 6a). However, on protein level nuclear expression of RB1 was significantly decreased in ResA cells, whereas E2F1 was significantly elevated in ResB cells (Fig. 6c). Since MYC is known to enhance the effect of E2F1^32^, we were interested whether the elevated Myc activity in ResB cells (Fig. 4b) may also play an essential role in the growth of this cell line. As expected, siRNA mediated MYC knockdown led to a reduction in the proliferation of all cell lines, but the effect was significantly stronger in ResB cells compared to LNCaP (Fig. 6d, Supplementary Fig. 4c). Taken together, these findings suggest that deregulation of the MYC/E2F1/RB1 cell-cycle regulator axis by various means is a ubiquitous event in AR indifferent prostate cancer that is necessary to overcome the G1 arrest caused by low AR activity.

## Discussion

The development of more potent second-generation anti-androgens has led to an increase in non-neuroendocrine tumours that are independent from AR pathway activity^23^. However, the molecular adaptations that lead to this sub-type of CRPC remain elusive due to the lack of well-characterized models that faithfully recapitulate the development of this phenotype. In this study, we describe the independent generation of two distinct anti-androgen resistant cell lines with an “AR indifferent” phenotype^24,25^. These cells express the AR and are partially androgen responsive at the transcriptional and proliferative level (Fig. 4). However, ResA and ResB cells show robust growth in presence of enzalutamide and during androgen deprivation (Fig. 1) although they have very low AR activity under these conditions (Fig. 4b). This AR-activity independent growth of ResA and ResB cells might also explain the low growth induction by low/intermediate R1881 levels since the cells already grow close to their maximum potential in androgen deprived medium (Figs 1b and 4d). In contrast, the enzalutamide and abiraterone resistant cell lines developed by Hoefer *et al*. and Puhr *et al*.^13,16^ have become resistant via mechanisms involving AR upregulation, induction of AR-V7 or GR dependent AR-bypass mechanisms, thus maintaining their dependency on AR/GR activity. Importantly, Puhr *et al*. found a strong induction of the GR in their enzalutamide resistant LNCaP cells^16^, which was not observed in the LNCaP derived ResA and ResB cell lines generated in this study (Supplementary Fig. 3d). This suggests that LNCaP cells can utilize a variety of resistance mechanisms to escape anti-androgen treatment by stochastic adaptation to the treatment rather than pre-determined cell type specific resistance mechanisms.

PCa cells with low AR activity have been associated with high self-renewal and tumour initiating capacity^26^ and both anti-androgen resistant cell lines had higher tumour take rates, an altered EMT signature, and formed more mero- and holoclones (Fig. 3), known to contain stem-like cells^27,28^. Due to their AR indifferent phenotype, ResA and ResB cells were highly resistant against all tested anti-androgens as well as androgen deprivation (Fig. 1a,b). Of note, the parental LNCaP cells were already partially resistant to the anti-androgen darolutamide due to the T877A mutation found in LNCaP^6^ and the CYP17 inhibitor abiraterone acetate was only active as a direct AR antagonist at high concentrations.

We observed that low anti-androgen concentrations increased the proliferation rate of ResB cells (Fig. 1a) in medium supplemented with androgen containing normal FCS, suggesting an anti-androgen withdrawal effect in ResB but not ResA cells^33,34^. Interestingly, enzalutamide on its own did not induce AR activity (Fig. 4b) in androgen deprived medium and the AR did not contain mutations that would convert enzalutamide into an agonist (Supplementary Fig. 3c). In this context it is essential to note that the biphasic androgen response^29^ was altered in ResB cells. They were strongly inhibited at androgen concentrations that led to optimal growth of LNCaP cells, whereas ResA cells showed no alteration in the dose dependency of the biphasic androgen response (Fig. 4d). We propose that low amounts of AR inhibitors can partially antagonize the androgen levels found in normal cell culture medium and thus might shift the AR activity from growth inhibitory to growth promoting levels in ResB cells. Of note, a subpopulation of anti-androgen resistant CRPC tumours has recently been demonstrated to be vulnerable to high testosterone concentrations in a clinical (phase II) trial^35^. The anti-androgen resistant cell lines developed in this study might be ideal models to study this innovative treatment option for late stage CRPC due to their difference in sensitivity.

Importantly, we confirmed the findings of van Soest *et al*.^11^ that enzalutamide treatment can confer docetaxel cross-resistance (Fig. 2a). This was caused by MCL1 mediated apoptosis resistance in the anti-androgen resistant cell lines (Fig. 2b,c), which corroborates that AR inactivation leads to MCL1 upregulation^36^. An additional mechanism of docetaxel resistance might be that the AR indifferent phenotype of the anti-androgen resistant cell lines confers insensitivity to docetaxel mediated AR inhibition^37^. AR signalling inhibitors and taxane based chemotherapies are the most commonly used systemic treatments for mCRPC^1^, which demonstrates the urgent need for novel therapeutic options for treatment induced cross-resistance. Of note, both anti-androgen resistant cell lines remained fully sensitive to treatment with the PARP inhibitor olaparib (causes synthetic lethality in cells with impaired DNA repair) and the pan BCL-2 inhibitor obatoclax (can overcome MCL1 mediated apoptosis resistance, Fig. 2d).

Anti-androgens induce G1 cell-cycle arrest in androgen dependent cells^38^, thus alterations in G1 checkpoint control may reduce AR dependency in cell-cycle progression. We found significant enrichment of the G1 associated cell-cycle control associated E2F and MYC target signatures in the anti-androgen resistant cell lines (Fig. 5b). Kumar *et al*. have previously found an inverse correlation between AR activity and E2F1 expression in mCRPC tissue samples^30^ and re-analysis of this dataset with the same gene sets used for the analysis of ResA and ResB cells confirmed an increased E2F target gene signature in mCRPC samples with low AR activity (Fig. 5c). Interestingly, knockdown of TP53/RB1 in LNCaP/AR also led to an AR independent basal-like phenotype^25^. In contrast, the group of Knudsen found that RB1 knockdown causes AR reactivation in a ligand independent manner^39^. Mechanistically, we did not identify copy number aberrations in E2F1/RB1 that might cause increased E2F activity in ResA and ResB cells (Fig. 6b), whereas Kumar *et al*. observed a high frequency of genomic RB1 deletions in their mCRPC dataset^30^. However, we found significant downregulation of cyclin D and upregulation of cyclin E in the anti-androgen resistant cell lines and mCRPC samples with low AR activity (Fig. 6a). Cyclin D-CDK4/6 is responsible for mono-phosphorylation of RB1, whereas cyclin E-CDK2 leads to RB1 hyper-phosphorylation^31^. Recently, Narasimha *et al*. have demonstrated that mono-phosphorylated RB1 is the active form that inhibits E2F1^31^, which suggests that the cyclin D/E alterations both lead to inactivation of RB1 and enhanced E2F activity. In addition, MYC activity was elevated in particular in the ResB cell line, which is known to enhance the transcriptional activity of E2F1^32^. MYC has been implicated in the development of enzalutamide resistance and increased Myc expression was correlated with shorter progression free survival in patients undergoing enzalutamide treatment^40–42^.

In summary, our findings suggest that the MYC/E2F1/RB1 cell-cycle regulator axis is deregulated by a large number of diverse mechanisms in PCa cells with low AR activity, which leads to high E2F activity. We propose that this compensates the lack of AR mediated growth signals and drives the proliferation of AR indifferent PCa cells.

## Materials and Methods

### Cell culture

The cell lines LNCaP-FGC and 22Rv1 were obtained from ATCC and cultured as recommended. The ResA cell line was generated by passaging of LNCaP cells with increasing concentrations of enzalutamide at the current IC_50_ value for 9.5 months whereas the ResB cell line was generated by continuous treatment with 10 µM RD-162 for 13 months. Importantly, both cell lines were generated independently from each other. Both cell lines were maintained in the same medium as LNCaP cells supplemented with 10 µM enzalutamide. The identity of LNCaP and both anti-androgen resistant cell lines was verified by comparison of the RNASeq data with the COSMIC database (Cell Lines Project v87)^43^ and mycoplasma test were performed at least once every 6 months. All experiments were performed in normal growth medium (containing 10 µM enzalutamide for ResA and ResB cells) unless otherwise noted.

### Proliferation measurements

The proliferation measurements were performed as described before^44^ by live cell analysis (IncuCyte ZOOM, Essen BioScience) using a nuclear mKate2 fluorescent label.

For proliferation experiments we used apalutamide (HY-16060), darolutamide (HY-16985), paclitaxel (HY-B0015), olaparib (HY-10162), and obatoclax (HY-10969) from MedChemExpress as well as enzalutamide (Sequoia Research Products, SRP016825m; and MedChemExpress, HY-70002), RD-162 (Sequoia Research Products), abiraterone acetate (Sequoia Research Products, SRP00371a), R1881 (Perkin Elmer, NLP005), and docetaxel (Fluka, 01885).

### Xenografts

Nude athymic mice (8–10 weeks old) were anesthetized with ketamine/xylazine and injected subcutaneously into each flank with 7.5 million cells suspended in matrigel (BD Biosciences, #356234). Once the tumour size reached ~200 mm^3^ the mice were treated with 10 mg/kg enzalutamide (dissolved in 1% Carboxymethyl cellulose, 0.1% Tween-80, and 10% DMSO) or vehicle by oral gavage on a daily basis. Tumour volume was monitored by calliper measurements every 4 days and the specific doubling rate was calculated by linear regression. The Ethical Committee for Animal Experimentation (ECD) of the KU Leuven approved all *in vivo* experiments (project number P078/2014) and the experiments were carried out in accordance with the relevant guidelines and regulations.

### Apoptosis measurements

We incubated the cells with the CellEvent Caspase-3/7 Green Detection Reagent (Thermo Fisher Scientific, C10723) and measured the percentage of positive nuclei 48 hours after treatment with the IncuCyte ZOOM live cell imaging system. For UV induced apoptosis, we irradiated the cells suspended in PBS in an UVLink 1000 crosslinker (Analytik Jena).

### Knockdown experiments

For siRNA mediated knockdown we transfected the cells with 30 pmol ON-TARGETplus SMARTpools (MYC: L-003282-02-0005; non-targeting control: D-001810-10) with Lipofectamine 3000 (Thermo Fisher Scientific, L3000015) and measured the proliferation rate as described.

### Morphology analysis

Images from three independent biological replicates were acquired on an IncuCyte ZOOM live cell imaging system and the outline of each cell was manually marked. The aspect ratio and circularity of each cell was measured with ImageJ.

### Spatial distribution analysis

Images from three independent biological replicates were acquired on an IncuCyte ZOOM live cell imaging system and the position of all cells was measured using a nuclear mKate2 fluorescent label. To determine the spatial distribution we determined how many cells are in close proximity (two nuclei diameters = 22 µm) to each cell. The data was summarized by calculating the average number of surrounding cells for each image and the total number of cells to correct for the cell density.

### High-resolution colony formation assays

We incubated 1500 cells per T75 flask (coated with poly-L-lysine hydrobromide) for 14 days and stained the colonies with 0.5% crystal violet in 25% methanol. The flasks were filled with pure white starch and scanned on a flatbed scanner (HP, Scanjet G4010) at 4800 dpi. The colonies were classified into para-, mero-, and holoclones with the CATCH Colonies software (v0.4, www.catch-colonies.net), which was verified by manual classification of 170 colonies under a microscope.

### Western blotting and subcellular fractionation

The cells were lysed in RIPA buffer supplemented with HALT protease inhibitor on ice and cleared by centrifugation for whole cell protein extraction. For subcellular fractionation the cells were resuspended in ice cold cytoplasmic lysis buffer (10 mM HEPES pH 7.9, 10 mM KCl, 1.5 mM MgCl_2_, 340 mM Sucrose, 10% Glycerol, 1 mM DTT, HALT protease inhibitor) before addition of 0.1% Triton X-100 and incubation for 5 minutes on ice. The nuclei were collected by centrifugation at 1300 RCF for 4 minutes at 4 °C and separated from the cytoplasmic proteins in the supernatant. The nuclei were washed once with ice-cold cytoplasmic lysis buffer before addition of sample buffer and sonication. The proteins were separated on NuPAGE Novex 4–12% Bis-Tris Protein Gels (Thermo Fisher Scientific) and transferred to 0.45 µm PVDF membranes (GE Healthcare, #10600023). The following antibodies were used: AR (in-house made), AR-V7 specific (Cell signaling, #68492), GAPDH (Merck Millipore, MAB374), H2AX (Cell signaling, #7631), MYC (Cell signaling, #5605), E2F1 (Santa Cruz, sc-193), and RB (BD, #554136). Detection was performed with an ImageQuant Las4000 using the western lightning Plus-ECL reagent (Perkin Elmer, NEL104001EA) and HRP conjugated secondary antibodies (DAKO, P0448 and P0260).

### Transcriptome analysis

Cells were seeded in DCC medium and after 24 hours they were treated with 10 nM DHT, 10 µM enzalutamide, a combination of the two, or vehicle (DMSO) for 18 hours. Total RNA was extracted with the RNAeasy plus mini kit (Qiagen, #74136) and RNA integrity was verified with an Agilent Bioanalyzer. The libraries for sequencing were generated with the TruSeq RNA Library Prep Kit according to the manufacturer’s recommendations and sequenced on a HiSeq 2500 System. The reads were mapped to the hg19 human reference genome using TopHat v1.3.3 with default settings. Differential gene expression and GSEA pathway analysis were performed with the Qlucore Omics Explorer v3.5. For analysis of mCRPC tissue samples a publicly available dataset generated by Kumar *et al*.^30^ was downloaded from the NCBI Gene Expression Omnibus (GEO) database (GSE77930). Gene set activity scores were calculated in R with the GSVA package^45^. For GSEA and GSVA analysis the Hallmark gene sets collection from the Molecular Signatures Database (MSigDB) was used. Unless otherwise noted all comparisons between the cell lines are at conditions similar to normal growth conditions (+DHT for LNCaP, +DHT and enzalutamide for ResA and ResB). All visualizations of the read coverage were performed with IGV^46^.

### Copy number variation (CNV) analysis

Genome-wide SNP genotyping was performed with Illumina CytoSNP arrays according to the manufactures recommendation and scanned on an iSCAN (Illumina). Genome-wide allele-specific copy number alterations were determined with ASCAT (v2.1)^47^. GSEA analysis of the “C1: positional gene sets” from MSigDB was used to identify cytogenetic bands with significant deregulation (FDR < 0.05) in the anti-androgen resistant cell lines compared to untreated LNCaP.

### Statistical analysis

R (v3.5.2) was used for the statistical analysis and all comparisons between two groups were done with Student’s t-Test. The Shapiro-Wilk Normality Test was used to confirm normal distribution of the samples in the xenograft experiment. P values lower than 0.05 were considered significant and indicated as follows: *P < 0.05, **P < 0.01, ***P < 0.001. All experiments have been performed in at least three biological replicates unless noted otherwise. Calculation of IC50 values was performed with the DRC package^48^ in R. All error bars represent the 95% confidence interval.

## Supplementary information

Supplementary Figures

Supplementary Table 1

## Data Availability

The RNA sequencing data generated in this study has been deposited at the NCBI Gene Expression Omnibus (GEO) website (https://www.ncbi.nlm.nih.gov/geo/) with the accession number GSE130534.
