# Supplementary material for: Drivers of AR indifferent anti-androgen resistance in prostate cancer cells
Source: Sci Rep. 2019 Sep 24;9:13786. doi: 10.1038/s41598-019-50220-1 (PMC6760229; doi:10.1038/s41598-019-50220-1)
Supplement: Supplementary file 1 — Supplementary Figures [file 41598_2019_50220_MOESM1_ESM.pdf]

Supplementary Figures

Drivers of AR indifferent anti-androgen resistance in prostate cancer cells

Authors
Florian Handle, Stefan Prekovic, Christine Helsen, Thomas Van den Broeck, Elien Smeets, Lisa Moris, Roy Eerlings, Sarah El Kharraz, Alfonso Urbanucci, Ian G. Mills, Steven Joniau, Gerhardt Attard, and Frank Claessens

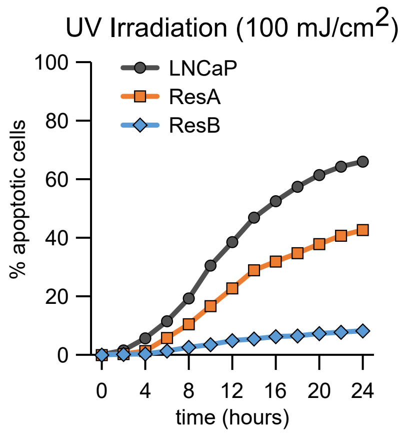

Supplementary Figure 1
The anti-androgen resistant cell lines have a high apoptosis resistance. Caspase 3/7 activity assays showing the percentage of apoptotic cells upon UV irradiation with 100 mJ/cm2 in the different cell lines in normal growth medium (containing 10 µM enzalutamide for ResA/ResB) over time (N=1).

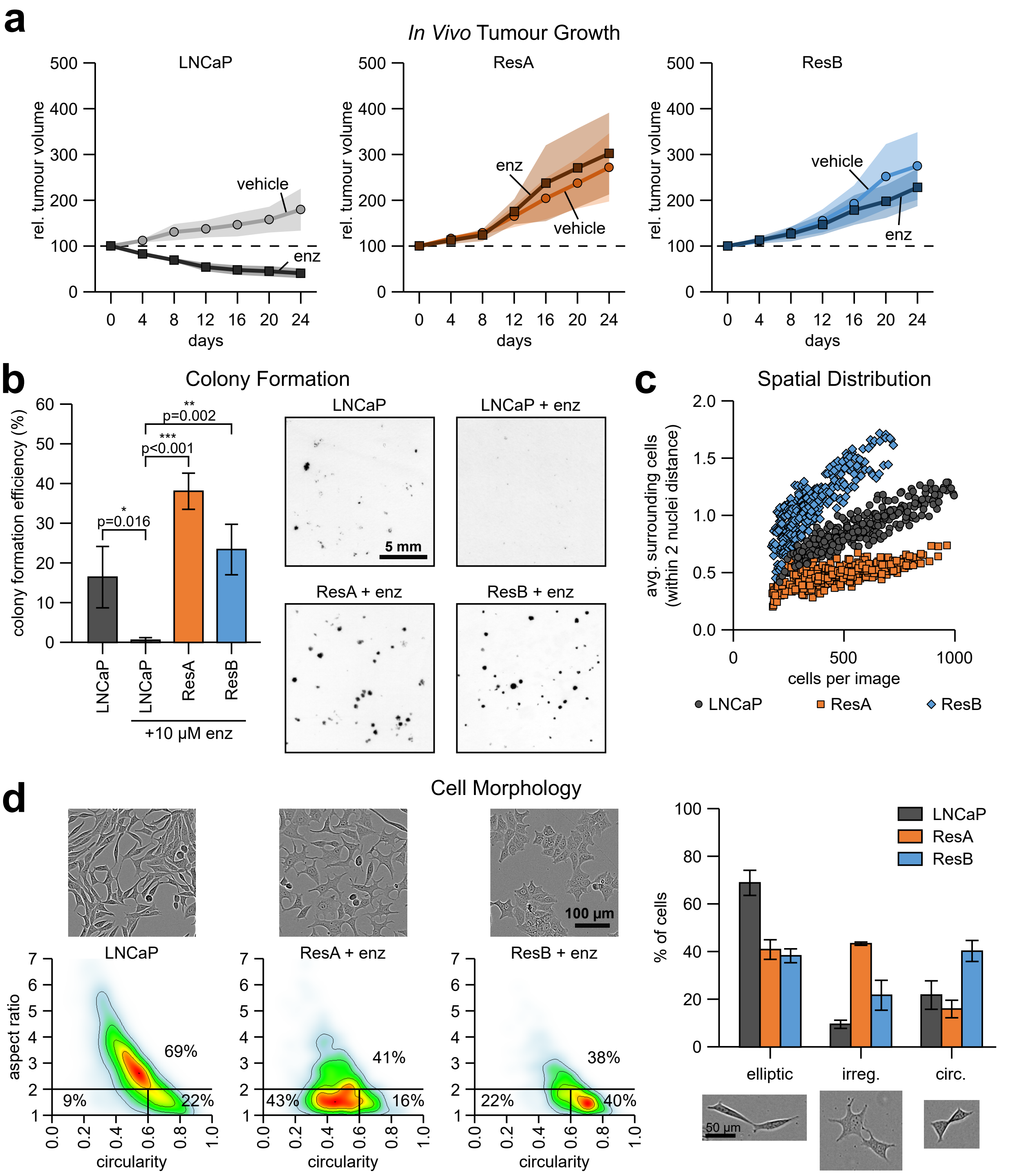

Supplementary Figure 2

Generation and characterization of the anti-androgen resistant cell lines. (**a**) In vivo tumor volume of xenografts derived from normal and anti-androgen resistant LNCaP cells in male mice treated with 10 mg/kg Enzalutamide or vehicle. The volume of each tumor was normalized to the volume at day 0 and set to 100 (dashed line). The solid line represents the average and the shaded areas indicate the 95% confidence interval. (**b**) Clonogenic assays showing the colony formation efficiency and representative images. (**c**) Spatial analysis of the cell distribution from three independent biological replicates showing the average number of cells that are in close proximity (2 nuclei diameter, approx. 22 µm) to each cell. Each dot represents one analyzed image. (**d**) Morphological analysis of >1000 cells per cell line from three independent biological replicates in normal growth medium (containing 10 µM enzalutamide for ResA/ResB). The cells were classified as elliptic, irregular, and circular based on their aspect ratio and circularity. The bar graph shows the average percentage of each morphology class as well as representative images for each type of cell morphology. All error bars represent the 95% confidence interval.

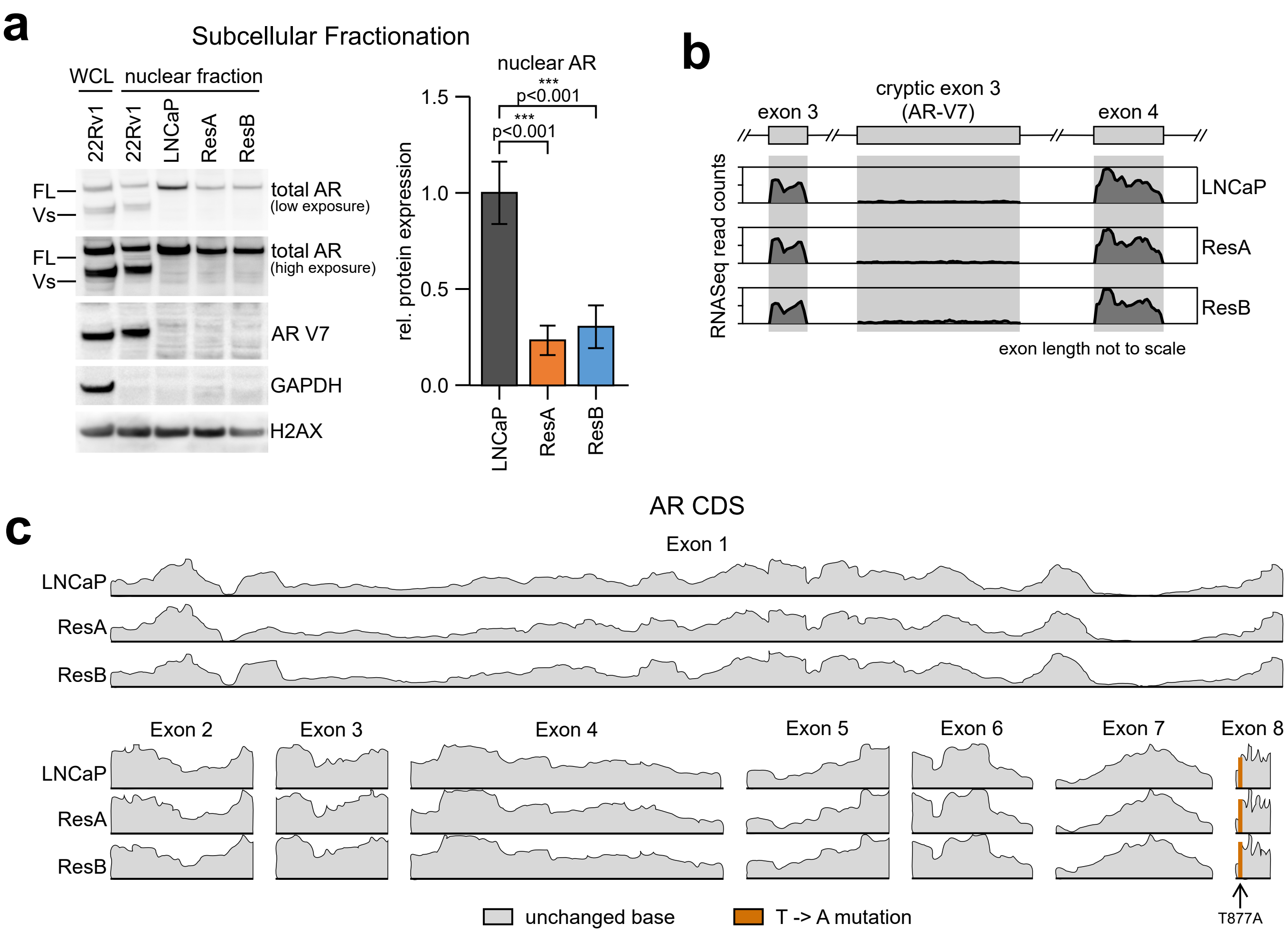

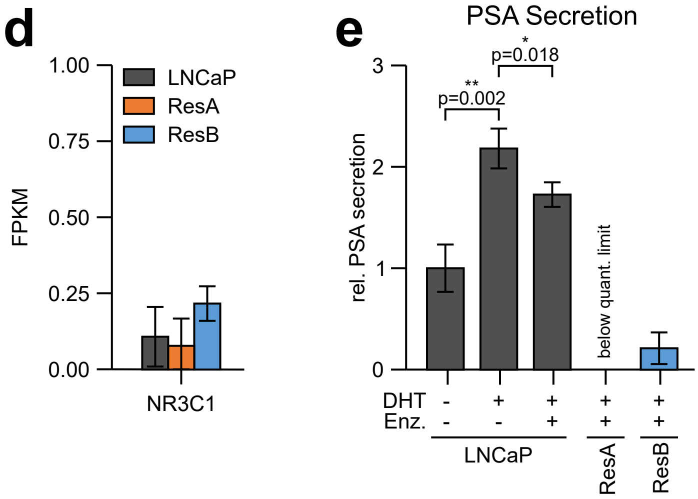

Supplementary Figure 3
Both anti-androgen resistant cell lines have an AR indifferent non-neuroendocrine phenotype. (**a**) Nuclear full-length (FL) and splice-variant (Vs) AR protein levels in normal growth medium (containing 10 µM enzalutamide for ResA/ResB) was measured by sub-cellular fractionation and western blotting. The uncropped blots are shown in Supplementary Figure 5. (**b**) RNASeq reads mapping to the V7 specific cryptic exon and the surrounding canonical exons. (**c**) RNASeq reads mapping to the coding sequence (CDS) of the AR. All bases that differ from the reference sequence in more than 20 % of the reads were colored, and all other positions are shown in gray. The location of the known LNCaP T877A mutation is indicated with an arrow. (**d**) FPKM (fragments per kilobase million) mRNA expression of the glucocorticoid receptor (NR3C1). (**e**) PSA protein secretion was measured by ELISA after treatment of the cells with 10 nM DHT, 10 µM enzalutamide (enz), or vehicle for 18 hours. All error bars represent the 95% confidence interval.

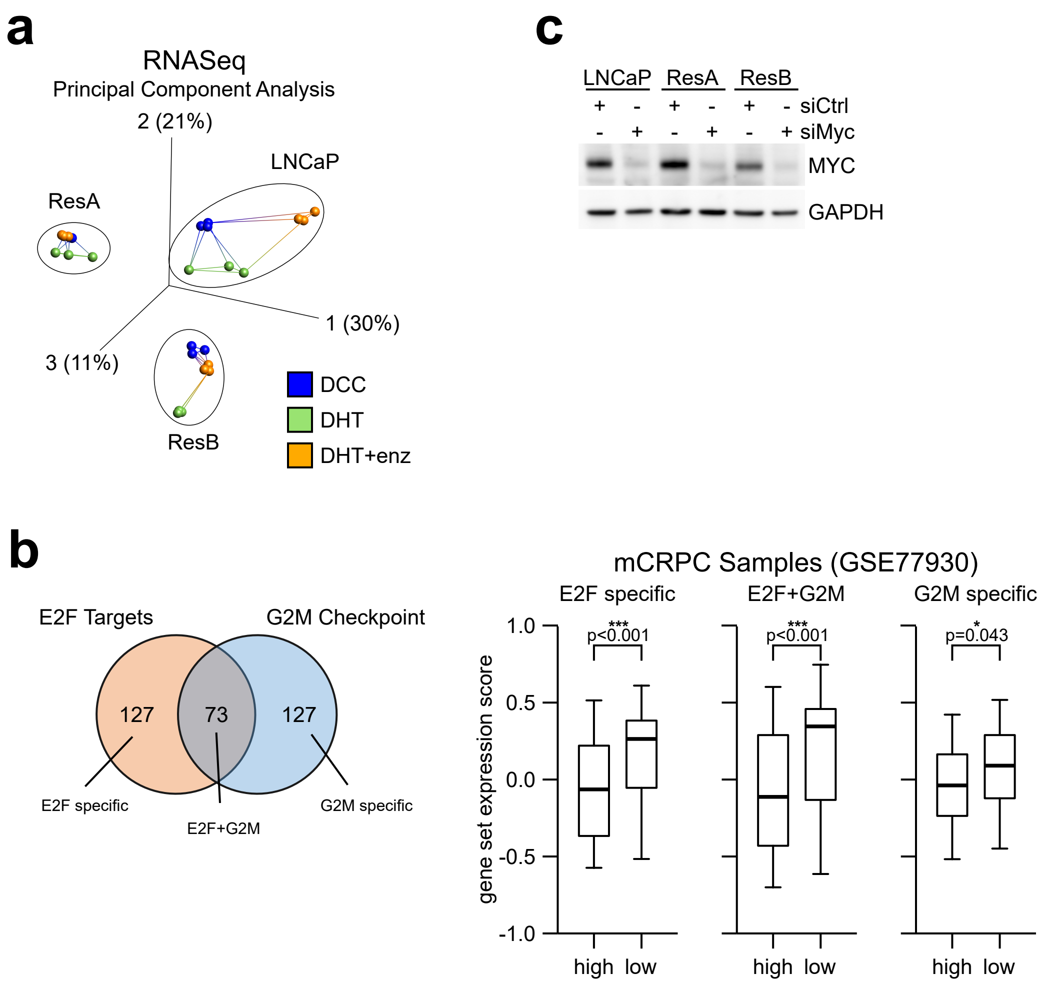

Supplementary Figure 4

Alterations on transcriptome level in AR indifferent PCa. (**a**) Clustering of the cell lines in Principal Component Analysis of the RNASeq datasets. (**b**) Venn diagram of the Hallmark "E2F targets" and "G2M checkpoint" signatures showing the overlap between the two datasets and GSVA gene set enrichment analysis of the unique and shared genes between the two datasets in the publicly available GEO dataset GSE77930 stratified by unsupervised clustering into samples with high and low AR activity. (**c**) Western blot showing the siRNA mediated MYC knockdown efficiency. The uncropped blots are shown in Supplementary Figure 5.

Full blots for Western Blot shown in Figure 4

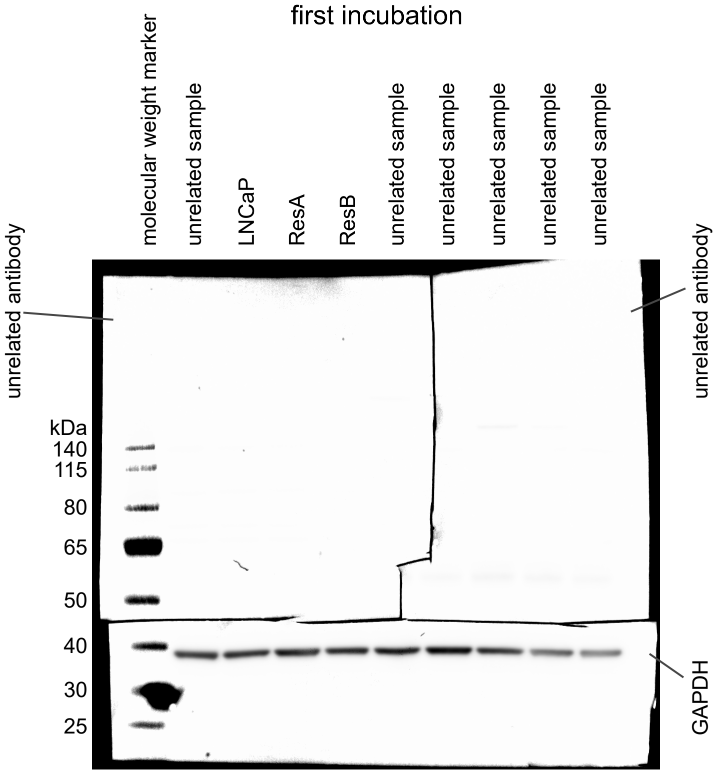

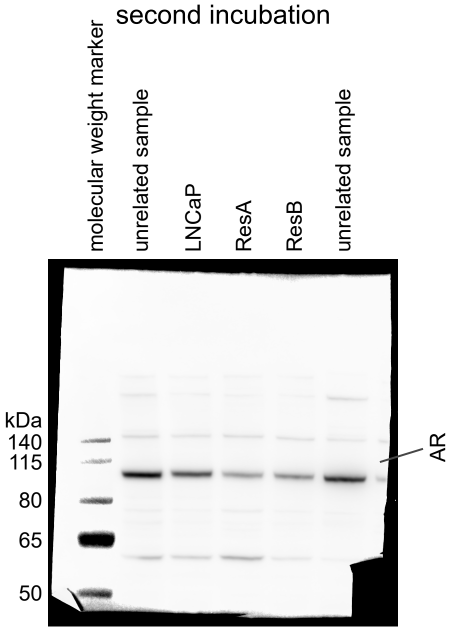

Supplementary Figure 5, continued on next page

Full blots for Western Blot shown in Figure 6

first incubation

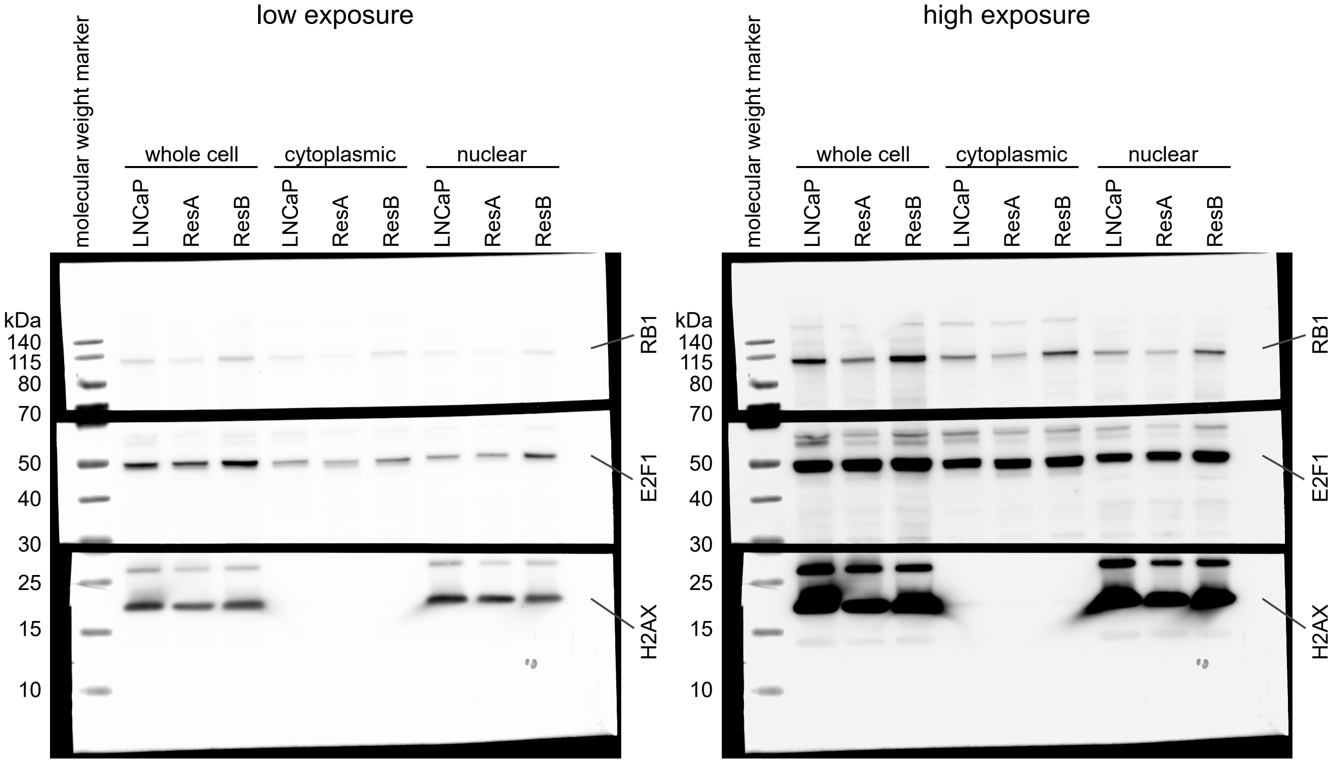

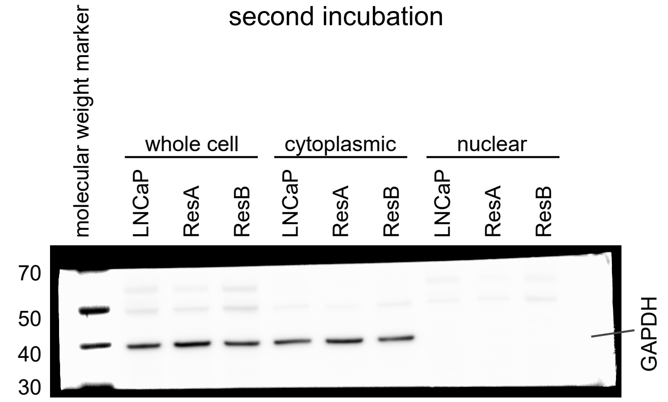

Supplementary Figure 5, continued on next page

Full blots for Western Blot shown in Supplementary Figure 3

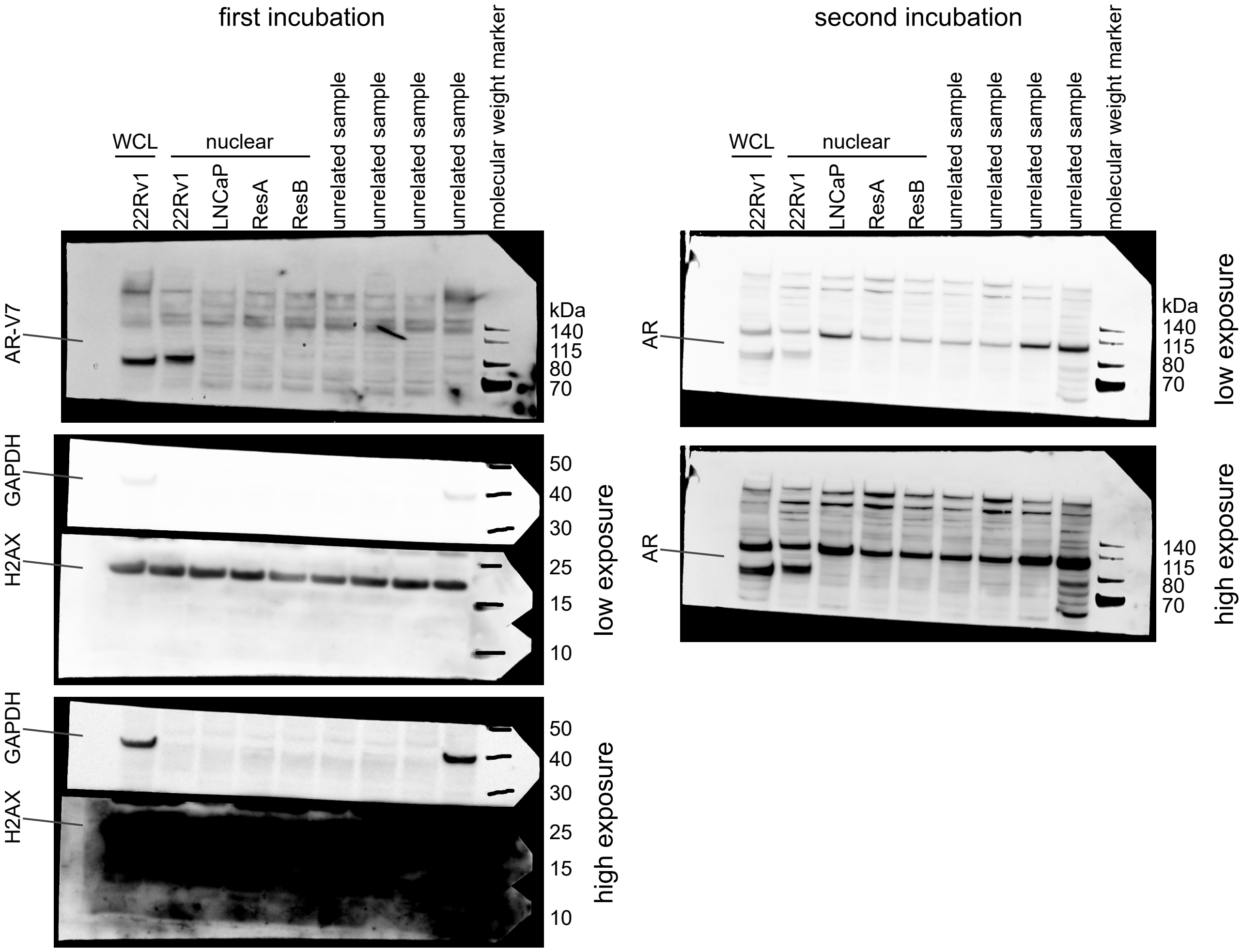

Supplementary Figure 5, continued on next page

Full blots for Western Blot shown in Supplementary Figure 4

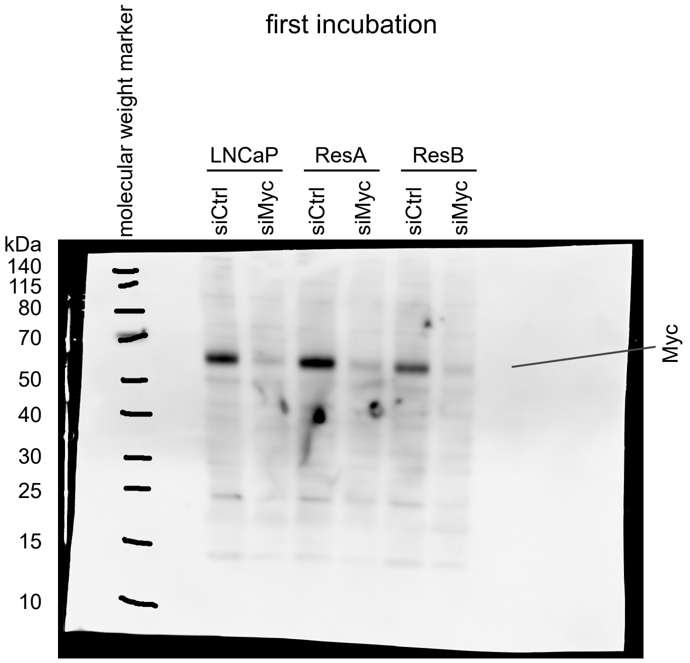

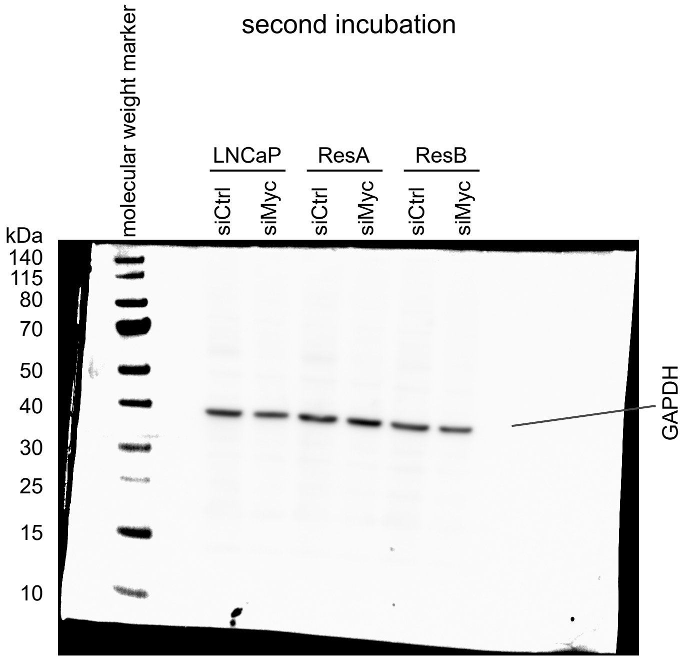

Supplementary Figure 5

Full uncropped western blots shown in this manuscript. The molecular weight marker is overlaid on all images.
